# Deaf and hard-of-hearing patients are unsatisfied with and avoid German health care: Results from an online survey in German Sign Language

**DOI:** 10.1186/s12889-023-16924-w

**Published:** 2023-10-18

**Authors:** Julia Rannefeld, Julie Lorraine O’Sullivan, Adelheid Kuhlmey, Jan Cornelius Zoellick

**Affiliations:** Charité – Universitätsmedizin Berlin, corporate member of Freie Universität Berlin and Humboldt-Universität zu Berlin, Institute of Medical Sociology and Rehabilitation Science, Charitéplatz 1, 10117 Berlin, Germany

**Keywords:** Deafness, Hearing loss, Persons with hearing impairments, Health services accessibility, Patient acceptance of health care, Right to health, Sign language, Surveys and questionnaires, Communication barriers

## Abstract

**Background:**

Approximately 235,000 deaf and hard of hearing (DHH) people live in Germany. Due to communication barriers, medical care for this group is difficult in many respects. Especially in the case of acute illnesses, the possibilities of communication, e.g., through sign language interpreters, are limited. This study investigates the satisfaction of DHH patients with medical care in Germany in unplanned medical consultations. The aim of this study is to provide insights into DHH patient's perception of medical care, to identify barriers and avoidance behaviours that stem from fears, miscommunication, and prior experiences.

**Methods:**

We obtained data from adult DHH participants between February and April 2022 throughout Germany via an online survey in German Sign Language. The responses of *N* = 383 participants (65% female, *M* = 44 years, *SD* = 12.70 years) were included in statistical analyses. Outcomes were convictions of receiving help, satisfaction with healthcare provision, and avoiding healthcare visits; further variables were concerns during healthcare visits, incidences of miscommunication, and a communication score. We calculated t-tests, ANOVAs, correlations, and linear and logistic regression analyses.

**Results:**

Our main findings show that (1) DHH patients were unsatisfied with provided healthcare (*M* = 3.88; *SD* = 2.34; range 0–10); (2) DHH patients reported many concerns primarily about communication and treatment aspects when visiting a doctor; and (3) 57% of participants deliberately avoided doctor visits even though they experienced symptoms. Factors such as concerns during doctor’s visits (B = -0.18; 95%CI: -0.34--0.02; *p* = .027) or miscommunication with medical staff (B = -0.19; 95%CI: -0.33–0.06; *p* = .006) were associated with satisfaction with medical care, while we found almost no associations with gender and location, and only few with age and education.

**Conclusions:**

Overall, our findings suggest that DHH patients are unsatisfied with provided healthcare, they deliberately avoid doctor visits, and they face various communication barriers. This study revealed several communication-related determinants of satisfaction with healthcare in DHH patients, such as incidences of miscommunication and the communication score. Communication-related barriers have high potential to be addressed in collaboration with the DHH community. To improve the medical care and the satisfaction with healthcare in DHH patients, training healthcare professionals, digital technologies, and other communication-enhancing interventions should be explored in future intervention studies.

**Supplementary Information:**

The online version contains supplementary material available at 10.1186/s12889-023-16924-w.

## Background

More than 1.5 billion people or 20% of the world’s population live with hearing loss [1]. The institution hospital and health care provision generally operate via sounds and thus heavily rely on acoustic signals to function. Calling the emergency service, hearing the call from the waiting room, understanding important instructions before examinations, or for taking medication are primarily and mostly exclusively delivered via spoken language. Communicating without sound as many deaf and hard-of-hearing (DHH) people do creates frictions with the established routines. Thus, many communicative situations in healthcare practices present barriers for DHH patients. This mismatch between institutional functioning and people’s hearing abilities impairs patients’ potential to receive the care needed. Disadvantages for DHH patients have been demonstrated regarding limited access to healthcare, and higher rates of misunderstandings of diagnoses and treatment [2–5]. This can lead to increased insecurities and both worse treatment outcomes and health in general compared to people without hearing loss, as well as lower levels of health literacy as a result of barriers to healthcare [2–4, 6]. Language differences are often cited as a cause of impeded communication between DHH patients and healthcare providers [7, 8]. DHH patients regularly report that they do not feel valued in the doctor-patient relationship and doctors in their opinion often do not respect their efforts to participate in their health care [9]; in addition, opinions on how to communicate effectively differ DHH patients and their physicians [7, 10].

In Germany, out of a population of 84.4 million approximately 235,000 DHH people in a conservative calculation represent an under-researched and potentially underserved community [9]. In his 2010 dissertation and to our knowledge the only large survey based on an barrier-free questionnaire of DHH patients in the last decade, Höcker [10] identified fears, worries, and moderate satisfaction during doctor visits. Precisely, DHH patients reported feelings of dependency and helplessness, as well as experiences of disrespectful treatment in the health system. Since Höcker’s publication, novel technologies or mask requirements as a response to the COVID-19 pandemic have changed communication practices in the medical field, some of which make it difficult for DHH patients to use sign language and lip reading [11]. Transparent FFP2-masks are rare and expensive, leading to difficulties for DHH patients and DHH healthcare professionals [12].

Sign language interpreters (SLIs) are often used to bridge the communication gap between medical staff and DHH patients, and clinics are legally required to provide SLI services. However, SLIs are best available for scheduled consultations and they are less accessible in unplanned or short-term medical visits as well as emergencies [13]. Additionally, medical staff and providers often believe that adequate communication is possible through lip-reading and written language deeming SLIs not necessary [14]. As a result, accompanying family members or friends often compensate this shortcoming, but since they are not certified, they cannot ensure factually and medically accurate translation [14, 15]. Incidences of misunderstanding, lower satisfaction with medical provision, and ultimately avoiding medical consultations might result from these experiences.

The aim of this article is to explore DHH patients’ experience in German healthcare settings with particular focus on unplanned consultations. To this end, we conducted an exploratory online survey in German Sign Language (GSL) including satisfaction with, concerns about, or avoiding healthcare provision with a convenience sample of DHH patients in Germany.

### Research questions and hypotheses

We formulated the following hypothesis: DHH patients report low levels of satisfaction with health care provision during unplanned medical visits (*M* ≤ 5.00; scale range 0–10). Additionally, we formulated the following exploratory research questions: 1) What are concerns of DHH patients about unplanned medical visits and how do they influence satisfaction? 2) What are barriers for DHH patients seeking health care consultations? 3) What sociodemographic and interactional variables are associated with satisfaction, help seeking, and convictions to receive help in DHH patients?

## Methods

### Study design

We collected data via an online questionnaire between 1 February and 18 April 2022 in written text and videos in GSL translated by a certified SLI. The questionnaire was administered via SoSci Survey v3.2.55 hosted on Charité in-house servers in accordance with European data protection laws. We pretested the survey twice with a female deaf participant and a female SLI, and we made improvements to wording and questions together. Many questions of our questionnaire were adapted from Höcker et al. [10] who in turn developed their questions from qualitative interviews with DHH people in a participatory fashion. Participants were recruited via emails to DHH associations or community multipliers and via social media channels.

### Measures and definitions

The questionnaire contained 49 items. The original German version and its English translation can be found in the supplementary material.

We collected sociodemographic data for age, gender (male, female, diverse), residential environment (rural, urban) and educational background (highest degree). Educational background was dichotomised into vocational training (0) and university degree [1]. This established classification follows the German education system, and it represents the two common paths of further education after leaving secondary school.

For the following measures, we used the percentage of maximum possible (POMP) score as a linear transformation using the formula “((x_i_—min)/(max—min)) * 10” where *x*_*i*_ are the individual observed values, *min* is the scale’s minimum value and *max* is the scale’s maximum value [16]. The resulting POMP score ranges between 0 and 10 and is thus intuitively interpretable in descriptive statistics and regression analyses.

*A communication score* (CS) was calculated with four 4-point Likert-scale items that included literacy in reading and writing words, understanding, and speaking spoken language. Responses ranged between *very badly* [1] and *very well* [4]. Following a principal component analysis with one factor (Eigenvalue = 2.25, 56% variance explained), we calculated the mean across the four items and transformed it into a POMP score [16] with a range between *low CS* (0) and *high CS* [10]. The scale had an internal consistency of Cronbach’s α = 0.74.

*Conviction to receive help in acute medical situations* (HELP) was measured by asking “Do you feel you get adequate help in medical emergency situations? “ on a 4-point Likert scale ranging from *strongly disagree* [1] to *strongly agree* [4], transformed into a POMP score ranging from *strongly disagree* (0) to *strongly agree* [10]. Medical emergency situations were specified to include all situations in which someone needs unplanned medical help; this includes unplanned visits, e.g., to the doctor, dentist, or hospital. All participants were asked this item regardless of their experience in emergency medical situations.

*Satisfaction during the latest doctor’s visit* (SAT) was measured with asking “Regarding your latest unplanned doctor’s visit, how satisfied were you with your appointment in general?” on a 4-point Likert scale ranging from *very dissatisfied* [1] to *very satisfied* [4], transformed into a POMP score ranging from *very dissatisfied* (0) to *very satisfied* [10]. Data were collected only from participants with previous emergency care experience (*n* = 245).

*Concerns during doctor visits* (CDV) were assessed via seven items e.g., being misunderstood or being misdiagnosed, on a scale from *not concerned at all* (0) to *very concerned* (100). Principal component analysis revealed one factor (Eigenvalue = 4.26, 61% variance explained). We calculated the mean across the seven items and transformed it into a POMP score with a range between *low CDV* (0) and *high CDV* [10]. The scale had an internal consistency of Cronbach’s α = 0.89. All participants were asked this item regardless of their experience in emergency medical situations.

*Miscommunication with medical staff* (MisC) was assessed via three 4-point Likert scale items on a scale from *totally disagree* [1] to *totally agree* [4] focusing on unasked questions in fear of being misunderstood, wrong diagnoses because of miscommunication, and feelings of helplessness and dependence because of deafness. Principal component analysis revealed one factor (Eigenvalue = 1.82, 61% variance explained). We calculated the mean across the four items and transformed it into a POMP score with a range between *low MisC* (0) and *high MisC* [10]. The scale had an internal consistency of Cronbach’s α = 0.67. Data were collected only from participants with previous emergency care experience (*n* = 245).

*Avoiding medical attention* (AMA) was assessed with the dummy variable (yes/no) whether participants did not use medical help even if they had symptoms indicating a condition. Subsequently, participants were asked to specify reasons for avoiding medical attention, e.g., fear of being misunderstood or not knowing where to go, with yes/no options and multiple responses.

### Inclusion criteria

We included DHH participants ≥ 18 years old living in Germany.

### Exclusion criteria

Participants younger than 18 years old and those without hearing impairment were immediately directed to the end of the survey without collecting any further data. After data collection, we excluded participants if they reported previous participations, if they only responded to ≤ 25% of questions, or if implausible time to response ratios were recorded [17].

### Sample size calculation

Following exchange with the Charité Institute for Biometry and Clinical Epidemiology, we set the minimum number of participants at *N* = 100 enabling linear regression analyses with five predictor variables.

### Statistical analysis

Data were analysed using IBM SPSS Version 27. We calculated descriptive statistics (mean and standard deviation), t-tests, ANOVAs, and correlations where applicable. We calculated two multivariable linear regression analyses to investigate individual factors (CS, CDV, MisC, age, gender, and location) associated with convictions of receiving help (HELP) and satisfaction with the last consultation (SAT), and a logistic regression analysis to investigate factors related to avoidance of medical attention (AMA). In logistic regression, we used backward elimination, eliminating non-significant predictors (*p* > 0.05) with every step. We calculated odds ratios (OR) and corresponding ninety-five percent confidence intervals (95%CI) for each predictor’s association with the outcome.

## Results

### Respondent characteristics

Overall,* n* = 578 respondents participated in the survey. We excluded *n* = 99 participants due to previous participations, *n* = 85 participants due to early dropouts, *n* = 7 participants due to implausible response time, *n* = 3 participants who did not specify hearing status, and *n* = 1 participant living outside of Germany.

After exclusion, *N* = 383 participants (65% female, *M* = 44 years, *SD* = 12.70 years) remained for statistical analysis. 265 participants (69%) reported being deaf, 91 participants (24%) were hard of hearing, and 27 participants (7%) were late-deafened. Seventy-six participants (20%) had a cochlea implant. In everyday life, participants communicated using GSL (53%), both GSL and spoken language in equal parts (25%), or mainly using spoken language (18%). Almost half of participants reported having at least one hearing family member fluent in both spoken language and GSL in their household (45%). Educational background among participants was high with 24% having obtained a university degree and 55% having completed vocational training. Most participants were employed full-time (45%) or part-time (23%). Household income was reported by most participants as 1,000–1,999€ per month (27%) or 2,000–2,999€ (22%) per month. Table 1 presents further sample characteristics.

**Table 1:** Sample characteristics

Variable	**Total**		**Female**		**Male**	
*N *(%)	383	100%	247	65%	126	33%
Age (M, SD)	44.26 (12.70)		42.57 (12.34)		47.77 (12.68)	
Hearing-impairment (N, %)						
Deaf	265	69%	167	68%	92	73%
Hard of hearing	91	24%	62	25%	27	21%
Late deafened	27	7%	18	7%	7	6%
Cochlea implant (N, %)	76	20%	57	23%	16	13%
Location (N, %)						
Urban	243	63%	157	64%	78	62%
Rural	94	25%	62	25%	30	24%
Educational background (N, %)						
University degree	93	24%	60	24%	30	24%
Vocational training	212	55%	138	56%	69	55%
Others	24	6%	13	5%	10	8%
Employment						
Full time employment	171	45%	93	38%	72	57%
Part time employment	88	23%	76	31%	10	8%
Student / pupil	24	6%	21	9%	3	2%
Pensioneer	36	9%	16	7%	20	16%
Other	43	11%	32	13%	9	7%
Not specified	45	12%	28	12%	17	14%
Household income						
No income	10	3%	7	3%	3	2%
Less than 1,000€ per month	46	12%	35	13%	10	8%
1,000–1,999€ per month	102	27%	73	30%	25	20%
2,000–2,999€ per month	64	22%	50	20%	33	17%
3,000€ or more per month	47	12%	21	8%	25	20%
Not specified	46	12%	31	13%	12	10%

*M* Mean, *SD* Standard deviation

### Communication score

Participants reported high scores in reading (*M* = 8.13; *SD* = 2.37) and writing (*M* = 7.57; *SD* = 2.41), but lower scores for understanding spoken language/lip-reading (*M* = 4.26; *SD* = 2.70) and for speaking spoken language (*M* = 5.08; *SD* = 3.30). Deaf participants reported lower CS (*M* = 5.62; *SD* = 1.81) than those who were deafened during their life course (*M* = 7.59; *SD* = 1.66) or the hard-of-hearing (*M* = 7.74; *SD* = 1.75) *(F*(2,379) = 55.97; *p* < 0.001; *d* = 1.19). Participants with cochlea implant reported higher CS (*M* = 7.18; *SD* = 1.61) than those without (*M* = 6.03; *SD* = 2.07) *(t(144*) = 5.22; *p* < 0.001; *d* = 0.58).

### Satisfaction, miscommunication, and concerns

Participants reported low to medium confidence they would receive help in acute medical situations (HELP; *M* = 4.48; *SD* = 2.46). They also reported various concerns during doctor’s visits (CDV; *M* = 6.30; *SD* = 2.45) with *being unable to understand healthcare professionals* as their strongest concern (*M* = 7.17; *SD* = 2.74). Those with experience in medical emergency situations (*n* = 245) reported frequent miscommunication with medical staff (*M* = 5.99; *SD* = 2.64) – *feeling helpless and dependent on the hearing* being their most frequent incident (*M* = 6.65; *SD* = 3.19). They also reported low levels of satisfaction with their previous doctor’s visits (*M* = 3.88; *SD* = 2.34; *t*(242) = -7.44, *p* < 0.001).

Table 2 shows correlations between the experiences, attitudes, communication score, and age. Results revealed medium to high relationships between the constructs. These relationships were largely independent of the participants’ age; solely incidences of miscommunication occurred more often for older participants.

**Table 2:** Two-tailed correlations (Pearson’s r) between main measurements

Variables	HELP	SAT	CS	CDV	MisC	Age
HELP	-					
SAT	.46^**^	-				
CS	.28^**^	.32^**^	-			
CDV	-.44^**^	-.36^**^	-.30^**^	-		
MisC	-.37^**^	-.32^**^	-.13^*^	.50^**^	-	
Age	-.02	.02	-.03	-.08	-.17^**^	-

211 ≤ *n* ≤ 308. *HELP* Conviction to receive help in acute medical situations, *SAT* Satisfaction with doctor’s visit, *CS* Communication score, *CDV* Concerns during doctor’s visit, *MisC* Miscommunication, *p < .05. **p < .01

No gender differences between men and women were found for the constructs in a series of t-tests (0.32 ≤|*t|*≤ 1.37; *p* ≥ 0.170).

For educational background, t-tests revealed that higher educated participants reported higher CS (*t*(303) = 5.40; *p* < 0.001; *d* = 0.67) and more confidence in receiving help (*t*(266) = 2.41; *p* = 0.017; *d* = 0.32).

Regarding location, t-tests showed that participants living in urban areas reported more confidence in receiving help (*t*(295) = 2.21, *p* = 0.01; *d* = 0.29).

Multiple linear regressions were calculated to investigate factors associated with HELP and SAT including CS, CDV, MisC, age, gender, location differences, and education. Table 3 displays results for the outcome HELP. The variables CS (B = 0.15; 95%CI: -0.00–0.30; *p* = 0.054), CDV (B = -0.24; 95%CI: -0.37–0.10; *p* = 0.001), MisC (B = -0.21; 95%CI: -0.34–0.09; *p* = 0.001), age (B = -0.03; 95%CI: -0.05–0.00; *p* = 0.020), and education (B = 0.61; 95%CI: 0.01–1.21; *p* = 0.07) were significant predictors for HELP.

**Table 3:** Multiple linear regression analysis predicting conviction to receive help in acute medical situations

						95% CI for B	
Variables	B	SE	β	T	p	LB	UB
constant	7.68	1.02		7.54	.000	5.68	9.69
CS	0.15	0.08	0.12	1.94	.054	-0.00	0.30
CDV	-0.24	0.07	-0.24	-3.38	.001	-0.37	-0.10
MisC	-0.21	0.06	-0.23	-3.45	.001	-0.34	-0.09
Age	-0.03	0.01	-0.14	-2.34	.020	-0.05	-0.00
Gender	-0.25	0.30	-0.05	-0.83	.406	-0.84	0.34
Location	-0.43	0.31	-0.08	-1.40	.163	-1.04	0.18
Education	0.61	0.31	0.12	2.00	.047	0.01	1.21

*N =* 247, Gender coded 0 male, 1 female, location coded 0 rural, 1 urban, education coded 0 vocational training 1 university degree, *SE* Standard error, *CI* Confidence interval, *R*^*2*^ = .26, corr. R^2^ = .24, *F*(7,239) = 11.89, *p* < .001

Table 4 shows results for the outcome SAT. The variables CS (B = 0.26; 95%CI: 0.09–0.43; *p* < 0.001), CDV (B = -0.18; 95%CI: -0.34–0.02; *p* = 0.027), and MisC (B = -0.19; 95%CI: -0.33--0.06; *p* = .006) were significant predictors for SAT.

**Table 4:** Multiple linear regression analysis predicting satisfaction during the latest doctor’s visit

							95% CI for B		
Variables	B		SE	β	T	p		LB	UB
constant	4.89		1.16		4.24	.000		2.61	7.17
CS	0.26		0.09	0.22	2.97	.003		0.09	0.43
CDV	-0.18		0.08	-0.18	-2.23	.027		-0.34	-0.02
MisC	-0.19		0.07	-0.22	-2.78	.006		-0.33	-0.06
Age	-0.01		0.01	-0.06	-0.86	.394		-0.04	0.02
Gender	0.24		0.35	0.05	0.68	.499		-0.46	0.94
Location	-0.13		0.35	-0.03	-0.36	.718		-0.81	0.56
Education	0.18		0.34	0.04	0.52	.524		0.60	-0.49

*N =* 183, gender coded 0 male, 1 female, location coded 0 rural, 1 urban, education coded 0 vocational training 1 university degree, *SE* Standard error, *CI* Confidence interval, *R*^*2*^ = .21, corr. *R*^*2*^ = .18, *F*(7,175) = 6.61, *p* < .001

### Avoiding medical care

The majority of participants (*n* = 218/383; 57%) reported they had not visited a doctor despite having symptoms indicating a medical condition. Most frequent reasons were communication barriers (*n* = 133/218, 61%), too much effort because of hearing disability (*n* = 97/218, 44%), and concerns of being misunderstood by the doctor (*n* = 52/218, 24%).

  We calculated a multiple logistic regression analysis to identify factors associated with avoiding medical attention seeking (yes/no) based on CS, CDV, MisC, age, gender, location, and education. Table 5 shows the results. CS, MisC, age, and education remained as significant predictors of avoiding medical attention in model 4 (backward stepwise modelling).

**Table 5:** Logistic regression analysis identifying factors associated with avoiding medical attention seeking

Variables	Model 1		Model 2		Model 3		Model 4	
	β (SE)	OR (95% CI)	β (SE)	OR (95% CI)	β (SE)	OR (95% CI)	β (SE)	OR (95% CI)
Constant	1.34 (.95)	3.81	1.56 (.91)	4.78	1.92 (.85)	6.85	1.44 (.79)	4.20
CS	-.17 (.07)	0.84 (0.73–0.97)	-0,17 (0.07)	0.84 (0.73–0.97)	-0,19 (0.07)	0.83 (0.72–0.96)	-0,18 (0.07)	0.84 (0.73–0.96)
MisC	.13 (.06)	1.14 (1.02–1.28)	0,13 (0.06)	1.14 (1.01–1.27)	0,16 (0.05)	1.17 (1.06–1.29)	0,16 (0.05)	1.17 (1.06–1.29)
Age	-.02 (.01)	0,98 (0.96–1.00)	-0,02 (.01)	0,98 (0.96–1.00)	-0,02 (.01)	0,98 (0.96–1.00)	-0,02 (.01)	0,98 (0.96–1.00)
Education	.67 (.31)	1,96 (1.07–3.60)	0,66 (0.31)	1,96 (1.06–3.57)	0,65 (0.31)	1,92 (1.05–3.52)	0,61 (0.31)	1,85 (1.02–3.36)
Location	-.53 (.30)	0,59 (0.33–1.07)	-0,53 (.30)	0,59 (0.33–1.07)	-0,51 (.30)	0,60 (0.34–1.08)		
CDV	.06 (.07)	1,07 (0.94–1.07)	0,07 (.07)	1,07 (0.95–1.22)				
Gender	.24 (.28)	1,27 (0.73–2.21)						
Nagelkerke R^2^	.150		.146		.141		.128	
Cox & Snell R^2^	.111		.108		.105		.095	
-2 Log likelihood	335.12		335.83		337.03		339.97	
Hosmer & Lemeshow	4.15		5.24		5.01		0.85	
N observed	272		272		272		272	
Chi-square	31.95^***^		31.23^***^		30.04^***^		27.10^***^	

*N =* 272, outcome avoidance coded 0 no, 1 yes, education coded 0 vocational training, 1 university degree, location coded 0 rural, 1 urban, gender coded 0 male, 1 female, *SE* Standard error, *OR* Odds ratio, *CI* Confidence interval, *p < .05; **p < .01; ***p < .001. Backward stepwise exclusion of non-significant predictors at α = .05 to arrive at the final model 4 with CS, MisC, age, and education as significant predictors for avoiding medical attention

## Discussion

This study focused on DHH patients’ self-reported experience and satisfaction with healthcare provision in Germany, particularly in unplanned medical consultations. Our main findings showed that [1] DHH patients were unsatisfied with provided healthcare; [2] DHH patients showed the biggest concerns in communication aspects followed by treatment aspects when visiting a doctor; and [3] DHH patients deliberately avoided doctor visits even though they experienced symptoms. This is particularly the case for older DHH patients with lower communication scores. Compared with Höcker’s [10] study on DHH patients’ experience from 2010, our results show little to no improvement in the perceptions of healthcare provision for this vulnerable group.

Correlations between the constructs CS, CDV, and MisC suggest a vicious cycle – patients with low communication scores experience more instances of miscommunication leading them to be more concerned about visiting doctors. In turn, those with more concerns and who have experienced miscommunication in medical settings are more likely to avoid medical care. Such a pattern complements previous findings on medical attention seeking all of whom agree on existing barriers in the fields of communication, health knowledge, and deaf cultural features [4, 10]. Sociodemographic variables showed only few relationships – gender and location did not produce any significant effects with our outcome measures; age and education were related with convictions to receive adequate help in acute medical situations (HELP) and with avoiding medical attention seeking (AMA). However, neither sociodemographic variable was related to satisfaction with the last doctor’s visit (SAT). In that sense, the communication barriers between the DHH patients and the audio-focused medical institutions seem to trump detrimental health effects for disadvantaged groups regularly reported [18–20] at least in the case of gender and location, and in some instances even for age and education. Studies usually report disadvantaged societal groups to use preventive healthcare services less frequently and to endure longer outpatient and inpatient treatment times [18–22]. Communication barriers between hearing patients and doctors resulting from an exclusive medical jargon have also been described particularly for disadvantaged patients [23].

However, studies looking at groups with comparable language barriers and socio-cultural differences have also shown – in line with our findings – that the influence of socio-demographic factors on satisfaction was low [24, 25]. Accordingly, access to healthcare often depends less on socio-economic factors, but rather on individual and cultural preferences or language and communication barriers [26, 27]. Language skills however seem to be an issue also for minority populations with negative effects on satisfaction with healthcare [24]. This supports the interpretation that frictions in communication – resulting from a different mother tongue and cultural background or hearing impairments – might be so influential that usually observed socio-demographic disadvantages lose their importance.

Breaking the vicious cycle through positive communicative experiences, such as available SLIs, time resources dedicated to understanding DHH patients’ concerns, health care professionals trained in the care and communication of DHH patients, or awareness of DHH people rights could increase DHH patients’ satisfaction and foster their trust in the medical system. Moreover, technological innovations that enable emergency calls in sign language or video calls with SLIs during an unscheduled medical consultation could also increase satisfaction and create more inclusive and accessible medical support.

Competent medical staff with sign language skills are urgently needed to achieve inclusive healthcare provision [10]. As long as there are hardly any sign language competent medical staff available, more sensibility training for dealing with DHH patients should be offered. These trainings have already been proposed [28], and including DHH people as consultants provides benefits such as better identification and elimination of inequalities [29]. Modern forms of delivering health information, such as text messaging or social media, have been used successfully in selected areas [30, 31]. Digital technology has the potential to bridge barriers, but requires reliable technology, access, and training [32].

We interpret low satisfaction ratings and consequent avoidance of medical attention as evidence for an underserved, vulnerable community affected by communication barriers because of a misfit with functional modes of healthcare institutions. However, as we have not surveyed people *without* hearing loss, we cannot directly compare those groups. Thus, dissatisfaction might also be the result of a generally underserving healthcare system. In contrast, a representative survey by a German insurance company showed a positive trend in satisfaction from 46% of participants being (very) satisfied in 2005 to 78% in 2021 [33]. In 2022 – the time of our data collection – satisfaction declined to 66% of participants being (very) satisfied, presumably as a result of the COVID pandemic. On the same scale, 32% of our participants reported being (very) satisfied with their latest unplanned doctor’s visit.

### Limitations

Our results are limited by the convenience sampling which is generally prone to selection bias. This generally impairs the representativeness of the sample. As a result, our study population is more highly educated and has a higher proportion of women, but it mirrors the age distribution of the general German population (*M* = 44.6 years) [34]. As male gender and lower education are associated with higher incidence of chronic diseases (coronary heart disease, diabetes) [35–37], the consequences of avoiding physician visits may be exacerbated in the overall DHH population compared with our selected sample. With our dichotomised operationalisation for educational status, we followed established practices of the German education system. We acknowledge other measures such as years of education [38] or CASMIN [39] that would have improved comparability with international studies. However, those measures limit the comparability to the German context as university degree versus vocational training presents the most common local distinction. We thus opted for national comparability limiting international comparability.

We enabled participants to apply the questions regarding their healthcare experience to different contexts, e.g., the emergency room, dentists, or general practitioners. This limits our ability to differentiate experiences in different contexts of healthcare provision, i.e., compare the emergency room to the dentist practice. Instead, we can only analyse the participants’ experience across contexts providing an insightful update of the general healthcare provision in unplanned medical situations as perceived by DHH patients. Further studies could differentiate the different contexts more and pay heed to contextual differences in an attempt to improve targeted healthcare provision.

Another limitation is the lack of a control group of non-hearing-impaired patients as our survey was aimed exclusively at DHH persons. Further studies might compare the two groups regarding satisfaction with and barriers of healthcare. Additionally, studies might also include insurance status of the participant particularly for the German context with the two-tier system between private and public insurance. As our survey was conducted during the COVID-19 pandemic, additional communication barriers for DHH patients (e.g., mandatory facial masks) potentially influence the low satisfaction, high concerns, and avoidance of doctor’s visits.

## Conclusions

Our study is among the first to address the existing research gap regarding healthcare of DHH patients in Germany. Most importantly, our results show a dissatisfaction of DHH patients with provided healthcare, concerns regarding both communication and treatment aspects, and avoidance of doctor visits. Especially older DHH patients with lower communication scores are at risk of receiving inadequate care. Our results show little impact of socio-demographic factors on satisfying healthcare provision for DHH patients. Thus, interventions (or prevention strategies, etc.) to improve medical care for DHH patients should focus on modifiable risk factors, such as educating medical staff to avoid miscommunication and misunderstandings. As our findings replicate many of the barriers and inequities reported in earlier studies [2, 7, 10, 13, 14, 40] we would like to stress the importance of adequate healthcare provision to vulnerable, underserved communities even in challenging times such as a global pandemic. Future studies in this area should include members of the DHH community to create awareness for their lived experiences in the medical context and beyond. Ultimately, this may present an opportunity to improve interactions of DHH patients with medical professionals and thereby increasing their trust and satisfaction with the healthcare system.

### Supplementary Information

**Additional file 1:** **Supplementary Table 1.** Questionnaire used in the online survey.

**Additional file 2:** **Supplementary Table 2.** Dataset for analyses.

**Additional file 3:** **Supplementary Table 3.** Overview approached organisations and social media accounts.

## Data Availability

All data generated or analysed during this study are included in this published article [and its supplementary information files].

## Abbreviations

DHH: Deaf or hard of hearing
SLI: Sign language interpreter
GSL: German sign language
POMP: Percentage of maximum possible
CS: Communication score
HELP: Conviction to receive help in acute medical situations
SAT: Satisfaction during the latest doctor’s visit
CDV: Concerns during doctor visits
MisC: Miscommunication with medical staff
AMA: Avoiding medical attention
