# Supplementary material for: Deaf and hard-of-hearing patients are unsatisfied with and avoid German health care: Results from an online survey in German Sign Language
Source: BMC Public Health. 2023 Oct 18;23:2026. doi: 10.1186/s12889-023-16924-w (PMC10583338; doi:10.1186/s12889-023-16924-w)
Supplement: Supplementary file 1 — Additional file 1: Supplementary Table 1. Questionnaire used in the online survey. [file 12889_2023_16924_MOESM1_ESM.pdf]

Supplementary table 1. Questionnaire used in the online survey

lfd Nr	Kennzeichnung/ labelling of variable	meaning of labelling	Fragentext	question translated to english	Ausprägung /value	Bedeutung	value label	Quelle/ referred to
0	teilnahme	participation	Ich habe schon einmal an der Befragung teilgenommen.	I have participated in the survey before.	1	ja	yes	
					2	nein	no	
					-1	keine Angabe	not specified	
1	hoerstatus	hearing_impairment	Sind Sie hörend, gehörlos/taub , spät ertaubt, schwerhörig?	Are you hearing, deaf/deafend, late deafend or hard of hearing?	1	hörend	hearing	
					2	gehörlos/taub	deaf	
					3	spät ertaubt	late deafend	
					4	schwerhörig	hrd of hearing	
					-1	keine Angabe	not specified	
					-9	nicht beantwortet	not answered	
2	cochlea	cochlea_implant	Tragen Sie ein Cochlea-Implantat (CI)?	Do you wear an cochlear implant (CI)?	1	ja	yes	
					2	nein	no	
					-1	keine Angabe	not specified	
					-9	nicht beantwortet	not answered	
3	taubspaet	late deafend	Wann sind Sie ertaubt?	When did you go deaf?		Ich war bei Ertaubung [] Jahre alt.	I was [] years old when I became deaf.	
					1	nicht gewählt	not selected	
					2	gewählt	selected	
4	gender	gender	Welchem Geschlecht fühlen Sie sich zugehörig?	Which gender do you feel you belong to?	1	männlich	male	
					2	weiblich	female	
					3	divers	diverse	
					-1	keine Angabe	not specified	
5	age	age	Wie alt sind Sie?	How old are you?		Alter in Jahren	age in years	
					-1	keine Angabe	not specified	
6	sprache_lesen	language_reading	Wie gut können Sie Wörter lesen?	How well can you read words?	1	gar nicht	very badly	nach (1)
					2	unsicher	bad	
					3	sicher	well	
					4	sehr sicher	very well	
					5	keine Angabe	not specified	
					-9	nicht beantwortet	not answered	
7	sprache_schreiben	language_writing	Wie gut können Sie schriftlich kommunizieren?	How well can you write words?	1	gar nicht	very badly	nach (1)
					2	unsicher	bad	
					3	sicher	well	
					4	sehr sicher	very well	
					5	keine Angabe	not specified	
					-9	nicht beantwortet	not answered	
8	sprache_laut_1	language_spoken_1	Wie gut können Sie Lautsprache verstehen oder Lippenablesen?	How well do you understand spoken language and/or lip-reading?	1	gar nicht	very badly	nach (1)
					2	unsicher	bad	
					3	sicher	well	
					4	sehr sicher	very well	
					5	keine Angabe	not specified	
					-9	nicht beantwortet	not answered	
9	sprache_laut_2	language_spoken_2	Wie gut können Sie Lautsprache sprechen?	How well can you speak spoken language?	1	gar nicht	very badly	nach (1)
					2	unsicher	bad	
					3	sicher	well	
					4	sehr sicher	very well	
					5	keine Angabe	not specified	
					-9	nicht beantwortet	not answered	
10	sprache_dgs_1	language_gsl_1	Wie gut können Sie in deutscher Gebärdensprache gebärden?	How well can you sign in German sign language?	1	gar nicht	very badly	
					2	unsicher	bad	
					3	sicher	well	
					4	sehr sicher	very well	
					5	keine Angabe	not specified	
					-9	nicht beantwortet	not answered	
11	sprache_dgs_2	language_gsl_2	Wie gut können Sie deutsche [illegible]	How well can you understand German sign language?	1	gar nicht	very badly	
					2	unsicher	bad	
					3	sicher	well	

	[illegible]	[illegible]	Gebärdensprache verstehen?	[illegible]	4	sehr sicher	very well	
					5	keine Angabe	not specified	
					-9	nicht beantwortet	not answered	
12	sprache_alltag	language_daily	Welche Sprache benutzen Sie im Alltag?	What language do you use in everyday life?	1	größtenteils in Deutscher Gebärdensprache (DGS)	mostly in German Sign Language (DGS)	adaptiert nach (2)
					2	grötenteils mit Lautsprachbegleitenden Gebärden (LBG)	mostly with spoken language accompanying signs (LBG)	
					3	zu gleichen Teilen in Gebärdensprache und in Lautsprache	in equal parts in sign language and spoken language	
					4	größtenteils in Lautsprache	mostly in spoken language	
					-1	keine Angabe	not specified	
					-9	nicht beantwortet	not answered	
13	sprache_famiilie	language_family	Haben Sie hörende Angehörige, die gut gebärden?	Do you have hearing relatives who sign well?	1	Ja, eine Person	Yes, one person	nach (1) #81
					2	Ja, zwei Personen	Yes, two persons	
					3	Ja, drei Personen	Yes, three persons	
					4	Ja, vier Personen oder mehr	Yes, four people or more	
					-1	keine Angabe	not specified	
					-9	nicht beantwortet	not answered	
14	erfahrung_yn	experience_yn	*In diese Umfrage wollen wir herausfinden, wie die Versorgung von Hörgeschädigten in Notfallsituationen erfolgt und empfunden wird. Hierzu gehören alle Situationen, in denen Sie ungeplant medizinische Hilfe brauchen, z.B. bei plötzlicher Atemnot. Dazu zählen aber auch ungeplante Besuche z.B. beim Arzt, Zahnarzt oder im Krankenhaus. Also Besuche, deren Grund eine akute neue Erkrankung waren. Haben Sie solche Erfahrungen bereits gemacht?*	In this survey we want to find out how the care of hearing-impaired people in emergency situations is provided and perceived. This includes all situations in which you need unplanned medical help, e.g. sudden breathing difficulties. But it also includes unplanned visits, e.g. to the doctor, dentist or hospital. In other words, visits for which the reason was an acute new illness. Have you already had such experiences?	1	ja	yes	
					2	nein	no	
					-1	keine Angabe	not specified	
					-9	nicht beantwortet	not answered	
15	zufrieden_arzt	SAT	Wenn Sie jetzt an Ihre letzten ungeplanten Arztbesuche zurückdenken: Wie zufrieden waren Sie insgesamt mit den Arztbesuchen?	Please think about your latest unplanned visitt doctor`s visit. How satisfied have you been with your appointment in general?	1	sehr unzufrieden	very dissatisfied	nach (1) #29
					2	unzufrieden	dissatisfied	
					3	zufrieden	satisfied	
					4	sehr zufrieden	very satisfied	
					-1	keine Angabe	not specified	
					-9	nicht beantwortet	not answered	
	help_know	help_know	Welche Einrichtung zur medizinischen Hilfe bei akuten Beschwerden außerhalb der Praxissprechzeiten (z.B. am Wochenende/ nachts) sind Ihnen bekannt?	What facilities are you aware of for medical help with acute complaints outside surgery hours (e.g. at weekends/at night)?	1	nicht gewählt	not selceted	
					2	gewählt	selceted	
16	help_know_1	help_know_1	Notruf 112	Emergency call 112				
	help_know_2	help_know_2	Notaufnahme	Emergency room				
	help_know_3	help_know_3	Kassenärztlicher Notdienst 116117	Emergency medical service hotline 116 117				
	help_know_4	help_know_4	Bereitschaftspraxen	On-call practices				
	help_know_other	help_know_other	sonstige	Other				
	help_know_kA	help_know_kA	keine Angabe	not specified				
	help_use	help_use	Welche dieser Einrichtungen haben Sie bei akuten Beschwerden schon mal genutzt?	Which of these facilities have you ever used for acute complaints?	1	nicht gewählt	not selceted	
					2	gewählt	selceted	
	help_use_1	help_use_1	Notruf 112	Emergency call 112				
	help_use_2	help_use_2	Notaufnahme	Emergency room				

17	help_use_3	help_use_3	Kassenärztlicher Notdienst 116117	Emergency medical service hotline 116 117				
	help_use_4	help_use_4	Bereitschaftspraxen	On-call practices				
	help_use_other	help_use_other	sonstige	Other				
	help_use_kA	help_use_kA	keine Angabe	not specified				
	call_know	call_know	Welche Möglichkeiten kennen Sie, den Notruf zu verständigen?	What ways do you know of to call 911?	1	nicht gewählt	not selceted	nach (3)
					2	gewählt	selceted	
18	call_know_1	call_know_1	Notfall-Telefax	Emergency fax				
	call_know_2	call_know_2	Nothilfe-SMS	Emergency SMS				
	call_know_3	call_know_3	Tess-Relaydienste	Tess relay services				
	call_know_4	call_know_4	Notruf-App	Emergency call app				
	call_know_5	call_know_5	Hilfe eines Hörenden	Help from a hearing person				
	call_know_6	call_know_6	andere	other				
	call_know_kA	call_know_kA	keine Angabe	not specified				
	call_use	call_use	Wenn Sie in den nächsten 5 Minuten einen Notruf verständigen müssten, was würden Sie nutzen?	If you had to call 911 in the next 5 minutes, what would you use?	1	nicht gewählt	not selceted	nach (3)
					2	gewählt	selceted	
19	call_use_1	call_use_1	Notfall-Telefax	Emergency fax				
	call_use_2	call_use_2	Nothilfe-SMS	Emergency SMS				
	call_use_3	call_use_3	Tess-Relaydienste	Tess relay services				
	call_use_4	call_use_4	Notruf-App	Emergency call app				
	call_use_5	call_use_5	Hilfe eines Hörenden	Help from a hearing person				
	call_use_6	call_use_6	andere	other				
	call_use_kA	call_use_kA	keine Angabe	not specified				
20	call_112_dgs	call_112_dgs	Wie wichtig ist es Ihnen, den Notruf in DGS absetzen zu können?	How important is it to you to be able to make the emergency call in DGS?	0	unwichtig	unimportant	
					100	sehr wichtig	very important	
					-1	keine Angabe	not specified	
					-9	nicht beantwortet	not answered	
21	nodoc_yn	AMA_yn	Sind Sie schon einmal nicht zum Arzt gegangen, obwohl Sie krank waren und einen Arzt gebraucht hätten?	Have you ever not visited a doctor, although you needed one?	1	ja	ja	
					2	nein	nein	
					-1	keine Angabe	not specified	
					-9	nicht beantwortet	not answered	
	nodoc_why_1-6	AMA_1-6	Falls ja, warum?	If yes, why?	1	nicht gewählt	not selceted	
					2	gewählt	selceted	
22	*nodoc_why_1*	*AMA_1*	*Weil ich Angst hatte, dass ich vom Arzt nicht verstanden werden*	*Because I was afraid, the doctor would not understand me*				
	nodoc_why_2	*AMA_2*	*Weil der Aufwand wegen meiner Hörschädigung zu groß war*	*Because the effort was too big due to my hearing impairment*				
	nodoc_why_3	*AMA_3*	*Weil ich nicht wusste, dass meine Krankheit schlimm sein könnte*	*Because I did not know, my medical condition could by harmful"*				
	nodoc_why_4	*AMA_4*	*Weil ich nicht wusste, an wen ich mich wenden soll*	*Because I did not know, where I could get help*				
	nodoc_why_5	*AMA_5*	*Weil ich durch die Kommunikationsbarriere nicht gerne zum Arzt gehe.*	*Because I do not like visiting doctor due to the communication barrier.*				
	nodoc_why_6	*AMA_6*	*anderer Grund*	*other*				
	nodoc_why_kA	AMA_na			-1	keine Angabe	not specified	
	feardoc	CDV	Wie groß sind die folgenden Sorgen für Sie, wenn Sie ungeplant zum Arzt müssen?	How major are following concerns when visitng a doctor unplanned.	0	sehr gering	concernded at all	
					100	sehr groß	very concerned	
					-1	keine Angabe	not specified	

					-9	nicht beantwortet	not answered	
23	*feardoc_1*	*CDV_1*	*Ich könnte keine dolmetschende Person finden*	*I might not be able to fing an SLI.*				
	feardoc_2	*CDV_2*	*Ich könnte das Gesundheitspersonal nicht verstehen*	*I might not be able to understand healthcare professionals.*				
	feardoc_3	*CDV_3*	*Ich könnte nicht verstanden werden*	*I might not being understood.*				
	feardoc_4	*CDV_4*	*Ich könnte ohne Einwilligung therapiert werden*	*I might be treated without giving consent.*				
	feardoc_5	*CDV_5*	*Ich kann vielleicht keine eigene Entscheidung treffen*	*I might not be able to make a decison on my own.*				
	feardoc_6	*CDV_6*	*Ich könnte falsch behandelt werden*	*I might be mistreated.*				
	feardoc_7	*CDV_7*	*Ich könnte eine falsche Diagnose bekommen*	*I might gat a false diagnosis.*				
	feardoc_8	*CDV_8*	*Sonstige:*	*other*				
24	help_ok	HELP	Haben Sie das Gefühl, in Notfallsituationen angemessene Hilfe zu bekommen?	Do you feel you get adequate help in medical emergency situations?	1	Stimme überhaupt nicht zu	strongly disagree	
					2	Stimme nicht zu	disagree	
					3	Stimme zu	agree	
					4	Stimme voll und ganz zu	strongly agree	
					-1	keine Angabe	not specified	
					-9	nicht beantwortet	not answered	
	arztbesuch	MisC	Wie sehr stimmen Sie folgenden Aussagen zu?	How much do you agree with the following statements?				
35	arztbesuch_1	MIsC_1	Ich habe schon einmal so getan, als hätte ich alles verstanden, obwohl ich noch Fragen hatte, weil ich nicht dumm aussehen oder dem Arzt lästig sein wollte.	I once already pretended I understood everything, though I still had questions, because I did not want to look dumb or be a burden for the doctor.	1	Stimme überhaupt nicht zu	strongly disagree	nach (1) #38 adaptiert
					2	Stimme nicht zu	disagree	
					3	Stimme zu	agree	
					4	Stimme voll und ganz zu	strongly agree	
					-1	keine Angabe	not specified	
					-9	nicht beantwortet	not answered	
36	arztbesuch_2	MIsC_2	Ich hatte bei einem ungeplantem Arztbesuch schon einmal das Gefühl, dass ich hilflos und abhängig von Hörenden bin.	At a previous unplanned medical appointment I felt helpless and dependent on hearing people	1	Stimme überhaupt nicht zu	strongly disagree	nach (1) #37 adaptiert
					2	Stimme nicht zu	disagree	
					3	Stimme zu	agree	
					4	Stimme voll und ganz zu	strongly agree	
					-1	keine Angabe	not specified	
					-9	nicht beantwortet	not answered	
37	arztbesuch_3	MIsC_3	Ich hatte schon einmal das Gefühl, dass eine falsche Diagnose gestellt wurde, weil der Arzt mich nicht verstanden hat.	I once already had the feeling of getting a false diagnosis, because the doctor did not understand me.	1	Stimme überhaupt nicht zu	strongly disagree	nach (1) #39 adaptiert
					2	Stimme nicht zu	disagree	
					3	Stimme zu	agree	
					4	Stimme voll und ganz zu	strongly agree	
					-1	keine Angabe	not specified	
					-9	nicht beantwortet	not answered	
38	arztbesuch_4	MisC_4	Ich hätte gerne eine Aufklärung über den Ablauf meiner Untersuchung oder Behandlung in deutscher Gebärdensprache, auch wenn keine dolmetschende Person anwesend ist (zB über fertige Videos)	I would like to be informed about the course of my examination or treatment in German sign language, even if no interpreter is present (e.g. via ready-made videos).	1	Stimme überhaupt nicht zu	strongly disagree	nach (1) #67
					2	Stimme nicht zu	disagree	
					3	Stimme zu	agree	
					4	Stimme voll und ganz zu	strongly agree	
					-1	keine Angabe	not specified	
					-9	nicht beantwortet	not answered	
39	Video_wichtig	video_importance	Wenn es eine Videoerklärung in DGS gäbe: Wie wichtig wäre es Ihnen eine Videoerklärung in DGS als Option zu haben, wenn Sie ungeplant zum Arzt müssen? *Videoerklärung über*	If there were a video explanation in DGS: How important would it be to you to have a video explanation in DGS as an option when you have to go to the doctor unplanned? Video explanation about planned examination or treatment	0	unwichtig	unimportant	
					100	sehr wichtig	very important	
					-1	keine Angabe	not specified	
					-9	nicht beantwortet	not answered	
40	Video_use	video_use	Wie wahrscheinlich wäre es, das Sie eine solche Videoerklärung in DGS nutzen würden?	How likely would you be to use such a video explanation in DGS?	0	unwahrscheinlich	unlikely	
					100	sehr wahrscheinlich	very likely	
					-1	keine Angabe	not specified	
					-9	nicht beantwortet	not answered	

			Jetzt stellen wir die noch ein paar Fragen, um die Hinergründe hinter der Versogung besser zu verstehen. Bitte beantworte Sie uns noch diese wenigen, letzten Fragen.	*Now we ask you a few more questions to better understand the reasons behind the supply. Please answer these few last questions.*				
41	Stadt_land	urban_rural	Wohnen Sie eher in einer Stadt oder auf dem Land?	Do you live more urban or rural?	1	Stadt	city	nach (1)
					2	Land	rural	
					-1	keine Angabe	not specified	
					-9	nicht beantwortet	not answered	
42	deutschland	germany	Wohnen Sie in Deutschland?	Do you live in Germany?	1	ja	yes	
					2	nein	no	
					-1	keine Angabe	not specified	
					-9	nicht beantwortet	not answered	
43	bundesland_1-16	state_1-16	In welchem Bundesland wohnen Sie?	In which federal state do you live?	1	Baden-Württemberg	Baden-Württemberg	
					2	Bayern	Bavaria	
					3	Berlin	Berlin	
					4	Brandenburg	Brandenburg	
					5	Bremen	Bremen	
					6	Hamburg	Hamburg	
					7	Hessen	Hesse	
					8	Mecklenburg-Vorpommern	Mecklenburg-Western Pomerania	
					9	Niedersachsen	Lower saxony	
					10	Nordrhein-Westfalen	Nordrhein-Westfalen	
					11	Rheinland-Pfalz	North Rhine-Westphalia	
					12	Saarland	Saarland	
					13	Sachsen	Saxony	
					14	Sachsen-Anhalt	Saxony-Anhalt	
					15	Schleswig-Holstein	Schleswig-Holstein	
					16	Thüringen	Thuringia	
					-1	keine Angabe	not specified	
					-9	nicht beantwortet	not answered	
44	land	country	In welchem Land wohnen Sie?	In which country do you live?	1	Österreich	Austria	
					2	Schweiz	Switzerland	
					3	andere EU-Staaten	other EU countries	
					4	außerhalb der EU	outside the EU	
					-1	keine Angabe	not specified	
					-9	nicht beantwortet	not answered	
45	abschluss	graduation	Welchen höchsten allgemeinbildenden Schulabschluss haben Sie?	What is your highest general school-leaving qualification?	1	ohne Abschluss (vorzeitig beendet)	without graduation (prematurely terminated)	
					2	Förderschule/ Sonderschule	Special school/ special education school	
					3	Volksschule/ Hauptschule	Primary school/ secondary modern	
					4	mittlere Reife/ Realschule (DDR: POS 10. Klasse)	Middle school leaving certificate/ Realschule (GDR: POS 10th grade)	
					5	Abitur/ Hochschulreife (DDR:	Abitur/ university entrance	
					6	noch Schüler	still a pupil	
					7	anderer Schulabschluss	Other school-leaving qualification	
					-1	keine Angabe	not specified	
	beruf	employment	Welcher Status der Erwerbstätigkeit trifft auf Sie zu?	Which employment status applies to you?	1	nicht gewählt	not selceted	nach (3)
					2	gewählt	selceted	
	beruf_1	employment_1	Vollzeit erwerbstätig	Full-time employed				
	beruf_2	employment_2	Teilzeit erwerbstätig	Working part-time				

46	beruf_3	employment_3	Altersteilzeit [unabhängig davon, ob in der Arbeit- oder Freistellungsphase befindlich]	Partial retirement [irrespective of whether in working or release phase]				
	beruf_4	employment_4	Geringfügig erwerbstätig, 400 Euro- oder Mini-Job	Marginally employed, 400 Euro or mini-job				
	beruf_5	employment_5	"Ein-Euro-Job" (bei Bezug von Arbeitslosengeld II)	One-Euro job (when receiving unemployment benefit II)				
	beruf_6	employment_6	Gelegentlich oder unregelmäßig	Occasionally or irregularly employed				
	beruf_7	employment_7	In einer beruflchen Ausbildung/Lehre	In vocational training/apprenticeship				
	beruf_8	employment_8	In Umschulung	In retraining				
	beruf_9	employment_9	Bundesfreiwilligendienst, Freiwilliges Soziales/Ökologisches Jahr	Federal voluntary service, voluntary social/ecological year				
	beruf_10	employment_10	Mutterschafts-, Erziehungsurlaub, Elternzeit oder sonstige Beurlaubung	Maternity or parental leave, parental leave or other leave of absence				
	beruf_11	employment_11	Schüler/Student	Pupil/student				
	beruf_12	employment_12	Arbeitslos	Unemployed				
	beruf_13	employment_13	Arbeitsunfähig	Unable to work				
	beruf_14	employment_14	Rentner	Pensioner				
	beruf_ka	employment_na	keine Angabe	Not specified				
47	ausbildung_1	education_1	Welchen höchsten beruflichen Abschluss haben Sie?	What is your highest professional qualification?	1	Lehre/Ausbildung	Vocational training	
	ausbildung_2	education_2			2	universitär	University	
	ausbildung_3	education_3			3	andere	other	
					-1	keine Angabe	not specified	
	ausbildung_kA	education_na			-9	nicht beantwortet	not answered	
48	finanzen	Income	Wie hoch ist ihr Haushalteinkommen im Jahr?	What is your household income per year?	1	Ich habe kein eigenes Einkommen	I have no income of my own	
					2	weniger als 250 € im Monat	less than 250 € per month	
					3	250 € bis unter 500 € im Monat	250 € to under 500 € per month	
					4	500 € bis unter 1000 € im Monat	500 € to under 1000 € per month	
					5	1000 € bis unter 1500 € im Monat	1000 € to under 1500 € per month	
					6	1500 € bis unter 2000 € im Monat	1500 € to under 2000 € per month	
					7	2000 € bis unter 2500 € im Monat	2000 € to under 2500 € per month	
					8	2500 € bis unter 3000 € im Monat	2500 € to under 3000 € per month	
					9	3000 € bis unter 3500 € im Monat	3000 € to under 3500 € per month	
					10	3500 € bis unter 4000 € im Monat	3500 € to under 4000 € per month	
					11	4000 € oder mehr im Monat	4000 € or more per month	
					12	keine Angabe	not specified	
					-9	nicht beantwortet	not answered	
49	Teilnahme	participation	Ich habe schon einmal an der Befragung teilgenommen.	I have taken part in the survey before.	1	ja		
					2	nein		
					-9	nicht beantwortet	not answered	
50	volljaehrig	volljaehrig	Sind Sie volljährig?	Are you of legal age?	1	Ich bin volljährig (18 Jahre oder älter).	Ich bin volljährig (18 Jahre oder älter).	
					2	Ich bin minderjährig (jünger als 18 Jahre).	Ich bin minderjährig (jünger als 18 Jahre).	2=Ausschluss
					-1	keine Angabe	not specified	
					-9	nicht beantwortet	not answered	0=Ausschluss

Note. This table includes all questions asked in the online survey, their value labels, and their variable names in the analyses (all in German original and with respective English translation). References for questions are as follows:

1 uni-mainz.de/bitstream/20.500 uni-mainz.de/bitstream/20.500.12030/1008/1/2456.pdf
2 ncbi.nlm.nih.gov/pmc/articles/I ncbi.nlm.nih.gov/pmc/articles/PMC6432988/
3 .gehoerlosen-bund.de/sachthe .gehoerlosen-bund.de/sachthemen/notruf
4 ts/MCRFileNodeServlet/DEMon ts/MCRFileNodeServlet/DEMonografie_derivate_00000210/1030817109004.pdf
