# Supplementary material for: Deaf and hard-of-hearing patients are unsatisfied with and avoid German health care: Results from an online survey in German Sign Language
Source: BMC Public Health. 2023 Oct 18;23:2026. doi: 10.1186/s12889-023-16924-w (PMC10583338; doi:10.1186/s12889-023-16924-w)
Supplement: Supplementary file 3 — Additional file 3: Supplementary Table 3. Overview approached organisations and social media accounts. [file 12889_2023_16924_MOESM3_ESM.pdf]

Supplementary table 3. Overview approached organisations and social media accounts

**Social media accounts**	
Werbevideo/ Studienlink via:	
Promotional video/ study link via:	
1	Instagram @umfrage_dgs
2	facebook: Umfrage-dgs.de
**Organisations**	
Angefragt, die Informationen über die Studie zu verteilen und um Teilnahme zu bitten, sowie Zusendung eines druckbaren Posters:	
Requested to distribute the information about the study and ask for participation, as well as sending a printable poster:	
1	Taubenschlag.de
2	Deutscher Gehörlosenbund e.V.
3	Landesverband der Gehörlosen BadenWürttemberg e.V.
4	Landesverband Bayern der Gehörlosen e.V.
5	Gehörlosenverband Berlin e.V.
6	Landesverband der Gehörlosen Brandenburg e.V.
7	Gehörlosenzentrum Bremen e.V.
8	Gehörlosenverband Hamburg e.V.
9	Landesverband der Gehörlosen Hessen e.V
10	Gehörlosen Landesverband Mecklenburg-Vorpommern e.V
11	Gehörlosenverein Ludwigslust e.V.
12	GV Niedersachsen eV
13	Landesverband der Gehörlosen Nordrhein-Westfalen e.V.
14	Landesverband der Gehörlosen Rheinland-Pfalz e.V.
15	Landesverband der Gehörlosen Saarland e.V.
16	Landesverband der Gehörlosen Sachsen e.V.
17	Gehörlosengemeinschaft Sachsen-Anhalt e.V.
18	Gehörlosen-Verband Schleswig-Holstein e.V.
19	Landesverband der Gehörlosen Thüringen e.V.
20	Diakonisches Werk Württemberg Beratung für Hörgeschädigte
21	Integrations- und Beratungszentrum Paderborn und Höxter gGmbH (IBZ gGmbH)
22	Bundesverband der Dozenten für Gebärdensprache (BDG)
23	Bundesverband der Gebärdensprach-dolmetscherInnen Deutschlands e.V.
24	Deutsche Arbeitsgemeinschaft für Evangelische Gehörlosenseelsorge e.V.

25	Gehörlosensportveband Nordrheinwestphalen
26	Deutscher Gehörlosen-Sportverband
27	Gehörlosen Landes Skat & Rommé Vereinigung NRW`93 e.V. (GLSRV NRW)
28	Deutsche Gehörlosen Skat- und Rommé Verband e.V.
29	Gesellschaft für Gebärdensprache und Kommunikation Gehörloser e.V.
30	Bundesvereinigung für Kultur und Geschichte Gehörloser e.V.
31	Netzwerk der Gehörlosen-Stadtverbände e.V.
32	Verband der Katholischen Gehörlosen Deutschlands e.V.
33	SAP HEAR and friends
34	nordtaube.de - Taube im Norden
35	Deutsche Gehörlosenzeitung
